# Macrophages in inflammatory multiple sclerosis lesions have an intermediate activation status

**DOI:** 10.1186/1742-2094-10-35

**Published:** 2013-03-04

**Authors:** Daphne YS Vogel, Elly JF Vereyken, Judith E Glim, Priscilla DAM Heijnen, Martina Moeton, Paul van der Valk, Sandra Amor, Charlotte E Teunissen, Jack van Horssen, Christine D Dijkstra

**Affiliations:** 1 Department of Molecular Cell Biology and Immunology, VU University Medical Center, Van der Boechhorststraat 7, BT Amsterdam, 1081, The Netherlands; 2 Department of Pathology, VU University Medical Center, De Boelelaan 1117, HV Amsterdam, 1081, The Netherlands; 3 Department of Neuroimmunlogy Unit, Blizard Institute, Barts and the London School of Medicine Dentistry, Queen Mary University of London, 4 Newark Street, London, E1 2AT, United Kingdom; 4 Department of Clinical Chemistry, VU University Medical Center, De Boelelaan 1117, HV Amsterdam, 1081, The Netherlands

**Keywords:** Multiple sclerosis, Macrophages, CD40, Mannose receptor

## Abstract

**Background:**

Macrophages play a dual role in multiple sclerosis (MS) pathology. They can exert neuroprotective and growth promoting effects but also contribute to tissue damage by production of inflammatory mediators. The effector function of macrophages is determined by the way they are activated. Stimulation of monocyte-derived macrophages *in vitro* with interferon-γ and lipopolysaccharide results in classically activated (CA/M1) macrophages, and activation with interleukin 4 induces alternatively activated (AA/M2) macrophages.

**Methods:**

For this study, the expression of a panel of typical M1 and M2 markers on human monocyte derived M1 and M2 macrophages was analyzed using flow cytometry. This revealed that CD40 and mannose receptor (MR) were the most distinctive markers for human M1 and M2 macrophages, respectively. Using a panel of M1 and M2 markers we next examined the activation status of macrophages/microglia in MS lesions, normal appearing white matter and healthy control samples.

**Results:**

Our data show that M1 markers, including CD40, CD86, CD64 and CD32 were abundantly expressed by microglia in normal appearing white matter and by activated microglia and macrophages throughout active demyelinating MS lesions. M2 markers, such as MR and CD163 were expressed by myelin-laden macrophages in active lesions and perivascular macrophages. Double staining with anti-CD40 and anti-MR revealed that approximately 70% of the CD40-positive macrophages in MS lesions also expressed MR, indicating that the majority of infiltrating macrophages and activated microglial cells display an intermediate activation status.

**Conclusions:**

Our findings show that, although macrophages in active MS lesions predominantly display M1 characteristics, a major subset of macrophages have an intermediate activation status.

## Introduction

Multiple sclerosis (MS) is a chronic inflammatory, demyelinating disease of the central nervous system (CNS). It is the most common cause of neurological disability among young adults, affecting approximately one in 1,000 individuals in Europe and North America [1]. The major pathological hallmarks of MS are multiple demyelinated lesions, which are associated with perivascular leukocyte infiltrates, astrogliosis, axonal damage and loss and neurodegeneration as well as remyelination [2,3]. Macrophages and activated microglia are abundantly present in demyelinating MS lesions and staging of MS lesions is based on the degree of myelin loss and the presence of human leukocyte antigens–-DR (HLA-DR) and CD68-positive macrophages [2].

Many lines of evidence indicate that macrophages play a dual role in the pathogenesis of MS as they contribute to lesion formation and axonal damage, but also support repair mechanisms [4,5]. Upon activation, macrophages secrete a plethora of pro-inflammatory mediators, such as cytokines, reactive oxygen species, nitric oxide and glutamate, which are able to induce tissue damage [6-11]. Injection of clodronate liposomes, which eliminate infiltrating macrophages, suppressed axonal damage and clinical signs of experimental autoimmune encephalomyelitis (EAE) [12,13], an animal model of MS, indicating that macrophages play an essential role in disease pathogenesis. However, the role of macrophages in the pathogenesis of MS is much more complex, since macrophages also exert beneficial effects. For example phagocytosis of myelin debris by macrophages/microglia is necessary for axonal sprouting and remyelination [12-22], and additionally, macrophages produce growth factors [23].

The dual role of macrophages can be explained by the fact that macrophages are not a single homogeneous population. Instead, several different phenotypical and functional subpopulations exist [24-26] as a result of their activation status, which is influenced by environmental signals [27-30]. The two most polarized phenotypes are classically activated (CA, M1) with cytotoxic and pro-inflammatory properties [29,30] and the alternatively activated (AA, M2) macrophages, which are involved in tissue repair by producing extracellular matrix molecules and anti-inflammatory cytokines [31,32]. An established method to generate M1 macrophages *in vitro* is by stimulation with interferon-γ (IFN-γ) and lipopolysaccharide (LPS) while induction of the M2 phenotype can be achieved by stimulation with IL-4 and many other stimuli [25,30,31]. Once induced *in vitro,* M1 and M2 macrophages can be distinguished by a panel of functional and phenotypical markers. *In vivo* the situation is more complex since a multitude of stimuli are present and markers are not exclusively expressed by M1 or M2 macrophages. The endogenous and environmental signals that determine the activation status are far more complex *in vivo* than *in vitro*, where time and dosage of the activating stimuli are selected. Numerous markers defining the phenotypes of M1 and M2 human macrophages have been described in the literature. These macrophage subsets are well studied in mouse models; however, the marker expression of the different subsets in humans are not completely consistent with findings in mice. For example, the most commonly used M1 markers of human macrophages include CD40 [32,33], CD86 [34], FcγRI (CD64) and FcγRII (CD32) [32,35], while mannose receptor (MR) [25,27,30] and CD163 [32,35,36] have been used to identify human M2 macrophages. In contrast, the most commonly used marker for M1 and M2 macrophages in mice are Nitric oxide synthase 2 and Arginase1 respectively [30].

To distinguish infiltrating monocytes from activated microglia a CCR2 red fluorescent protein knock-in mouse was recently reported in which infiltrating monocytes are red fluorescent while resident microglia are green fluorescent [37]. After EAE induction in this mouse the relative contribution of infiltrating monocytes and activated microglia could be distinguished at the level of pathology. In humans such discrimination between macrophages and microglia is not yet feasible. Macrophages/microlgia present in active and chronic active MS lesions contain lipids, reflecting ingestion and accumulation of myelin lipids. These so-called foamy macrophages express anti-inflammatory cytokines and lack typical pro-inflammatory cytokines, indicating an alternative phenotype [38,39]. On the other hand, it has been shown that macrophages in inflammatory MS lesions express specific M1 markers, such as inducible nitric oxide synthase (iNOS) and CD40 [40,41]. These data prompted us to systematically analyze the expression of a discriminatory panel of M1 and M2 markers in well-characterized MS lesions and normal appearing white matter (NAWM). Although foamy macrophages and microglia in active and chronic active lesions predominantly express M1 markers, the majority (approximately 70%) of CD40-positive macrophages also express the typical M2 marker MR. Taken together, our findings indicate that foamy macrophages in active demyelinating MS lesions display an intermediate activation status supporting the idea that *in vitro* polarization of macrophages and microglia cannot be easily translated to pathology of diseased tissues *in vivo*.

## Materials and methods

### Human brain tissue

Human brain tissue was obtained at autopsy from two patients without neurological disorders (control) and eight MS patients. Patient characteristics are listed in Table 1. The rapid autopsy regimen of the Netherlands Brain Bank in Amsterdam (coordinator Dr. I. Huitinga) was used to acquire the samples, with the approval of the Medical Ethical Committee of the VU University Medical Center. All patients and controls had given informed consent for autopsy and use of their brain tissue for research purposes. Tissue samples from subcortical white matter were obtained from non-neurological control cases. For MS tissue, the clinical diagnosis was confirmed neuropathologically by Professor P. van der Valk. Tissue samples from MS cases were obtained after *ex vivo* magnetic resonance imaging scanning as described by de Groot *et al*. [42]. Brain tissue samples were snap-frozen and stored in liquid nitrogen. Classification of lesion staging was based on immunohistochemical detection of inflammatory cells (that is, cells that express major histocompatibility complex (MHC) class II/HLA-DR) and the presence of proteolipid protein (PLP) to reveal areas of myelin loss or the presence of myelin in phagocytic cells as described before [43-45]. Seven lesions sampled in this study were classified as active lesions with myelin loss and abundant phagocytic perivascular and parenchymal macrophages containing myelin degradation products, and five lesions were classified as chronic active with a hypocellular demyelinated gliotic center with astrogliosis and a hypercellular rim containing activated microglia and macrophages.

**Table 1:** Patients’ details

**Case**	**Tissue block lesion characterization**	**Gender**	**Age**	**Disease duration (years)**	**Cause of death**	**PMD (hrs:min)**
Control		Male	74	-	Lung carcinoma	7:45
Control		Male	62	-	DM *de novo*, suspected pancreas carcinoma	7:20
MS	3 blocks active	Male	41	14	Urosepsis	7:23
MS	2 blocks active	Male	54	22	Euthanasia	8:15
MS	2 blocks active	Male	51	20	NA	11:00
MS	1 block chronic active	Female	66	23	NA	6:00
MS	1 block chronic active	Male	61	18	Euthanasia	6:00
MS	2 blocks chronic active	Male	66	26	Ileus	9:15
MS	1 block chronic active	Male	49	26	Pneumonia	7:30

DM, diabetes mellitus; NA, not applicable; PMD, postmortem delay time (hours: minutes).

### Consent

Brain tissue samples were obtained from the Netherlands Brain Bank (coordinator Dr. Huitinga, Amsterdam, The Netherlands).

The Netherlands Brain Bank received permission to perform autopsies for the use of tissue and for access to medical records for research purposes from the Ethical Committee of the VU University Medical Center, Amsterdam, The Netherlands.

All patients and controls, or their next of kin, had given informed consent for autopsy and use of brain tissue for research purposes.

### Human macrophages

**Figure 1:**
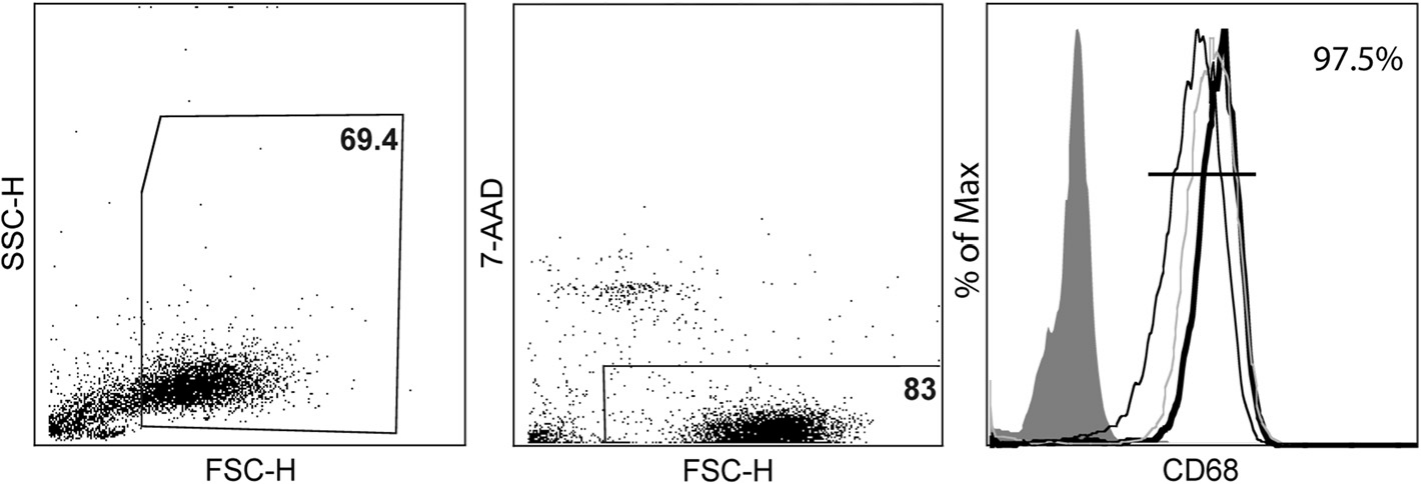
**Characterization of monocyte-derived macrophages.** Monocytes were isolated from peripheral blood mononuclear cells (PBMCs) of healthy donors and were cultured for 7 days in the presence of 5% normal human serum. A gate was positioned around living (7-aminoactinomycin (7-AAD) negative) cells (approximately 70%). Of the living cells approximately 97% stained positive for CD68. Representative fluorescence-activated cell sorting (FACS) plots are shown.

Peripheral blood mononuclear cells (PBMCs) were isolated from healthy donor buffy coats (Sanquin Blood Bank, Amsterdam, The Netherlands) using Ficoll (Lymphoprep™, Axis-Shield, Oslo, Norway) density gradient. Monocytes were isolated from the PBMCs by anti-CD14 magnetic beads according to manufactures protocol (Miltenyi Biotec, Leiden, The Netherlands). Monocytes were cultured in 6-well plates (Greiner Bio-One; Alphen a/d Rijn, The Netherlands) at a concentration of 1 × 10^6^ cells/ml in macrophage medium (DMEM (Invitrogen, Breda, the Netherlands), supplemented with 5% (v/v) normal human serum (NHS) (Bio Whittaker, East Rutherford, NJ), and 1% (v/v) penicillin-streptomycin-glutamine (Invitrogen), at 37°C, 5% CO_2_. Monocytes matured into macrophages (M0 macrophages) in the course of 5 to 7 days of culturing. Before each experiment macrophages were washed with phosphate buffered saline (PBS) (Braun, Melsungen, Germany), resulting in >95% pure macrophages cultures (Figure 1).

The M1 phenotype was induced by culturing M0 macrophages in the presence of 1 × 10^3^ U/ml recombinant human IFN-γ (U-Cytech, Utrecht, the Netherlands) [46] and 10 ng/ml *Escherichia coli* LPS (026:B6; Sigma-Aldrich, Zwijndrecht, the Netherlands) for 48 h. M2 macrophages were generated using 10 ng/ml human IL-4 (ImmunoTools, Friesoythe, Germany) for 48 h [25].

### Fluorescence-activated cell sorting (FACS) analysis

Cells were treated with 4% (v/v) lidocaine for 10 min, harvested and washed with PBS. Subsequently the cells were fixed with 4% formaldehyde for 30 min on ice, washed with PBS containing saponine 1% (v/v), 0.1% (v/v) bovine serum albumin (BSA). Next, cells were incubated with the first antibody directed against intracellular or cell surface markers (Table 2) diluted in PBS containing saponine 1% and 0.1% BSA for 1 h. After being washed twice, the cells were incubated with the fluorescently labeled secondary antibody ALEXA 488 goat anti-mouse (Invitrogen 1:400) for 1 h. As a control, cells were incubated with the isotype-matched IgG controls or anti-mouse IgG as a second antibody. The macrophages were analyzed using flow cytometry (FACSCalibur, Becton Dickinson, Erembodegem, Belgium) combined with Cellquest Pro software (Becton Dickinson) and FlowJo software version 9.4.0 for Microsoft (Tree Star, San Carlos, CA). Data obtained using fluorescence-activated cell sorting (FACS) analysis were presented as mean fluorescent intensity (MFI). The data from three separate experiments performed in duplicate were averaged and expressed as mean ± SEM.

**Table 2:** Antibodies used in fluorescence-activated cell sorting (FACS) analysis and immunohistochemistry (IHC)

**Antigen**	**Species**	**Dilution (FACS)**	**Dilution (IHC)**	**Double stain (IHC)**	**Manufacturer**
CD68 (KP1)	mouse	1:100	1:1000	1:1000	Dako
CD40 (Clone MCA1590)	mouse	1:50	1:1500	1:750	Serotec
CD86 (MCA1118)	mouse	1:50	1:400	-	Serotec
CD64 (555525)	mouse	1:50	1:250	-	Serotec
CD32 (MCA1075)	mouse	1:500	1:2000	-	Serotec
CD206 (Clone 19.2)	mouse	1:100	1:400	1:250 (bio 1:50)	BD Pharmingen
CD163 (ED1)	mouse	1:50	1:1000	-	Serotec
HLA-DR (LN3)	mouse	-	1:1000	1:1000	Dako

### Immunohistochemistry

Frozen sections of MS lesions, normal appearing white matter (NAWM) and normal control brain tissue were air dried and incubated with acetone for 10 min. Sections were rehydrated in PBS and pre-incubated with 10% NHS in PBS/0.1% BSA (Roche, Mannheim, Germany) for 60 min. Subsequently, sections were incubated with the appropriate primary antibody (Table 2) overnight at 4°C in PBS/0.1% BSA. After washing the sections were incubated with a secondary antibody with a Dako Envision Kit (Peroxidase) (Dako, Heverlee, Belgium) for 30 min at room temperature, rinsed in PBS stained with diaminobenzidine (DAB) (Dako) rinsed with tap water and counterstained with hematoxylin (Sigma-Aldrich). Finally, sections were dehydrated and embedded in Entellan (Merck, Schiphol-rijk, The Netherlands). Isotype controls were used as a negative control; however, omission of the primary antibody did not show any differences in specificity. Images were taken on a Nikon E800 microscope (Amstelveen, the Netherlands) and processed using Adobe Photoshop 6.0 (San Jose, USA).

Double staining was performed using anti-CD40, anti-MR-bio and anti-HLA-DR to determine the extent of colocalization of CD40 and MR with each other and with anti-HLA-DR as microglia/macrophage marker. Sections were fixed using acetone, pre-incubated with 10% NHS in PBS/0.1% BSA. Subsequently, sections were incubated with anti-CD40 or anti-MR overnight at 4°C. Next, sections were washed and incubated for 1 h with goat-anti-mouse IgG-Alexa 488 (Invitrogen, Breda, The Netherlands) or goat-anti-mouse IgG-Alexa 647. Sections were washed and incubated with either anti-CD40, anti-MR or anti-HLA-DR (clone LN3: eBioscience) in PBS/0.1% BSA for 1 h. They were then washed again and incubated with a goat-anti-mouse IgG1-Alexa 488 or goat-anti-mouse IgG2a Alexa 647 (Invitrogen) antibody. Sections were counterstained using Hoechst (Sigma-Aldrich) 1:10,000 for 5 min, rinsed and embedded using mounting medium. Images were taken on a Leica DM6000 (Leica LAS AF software, Leica Microsystems, Bensheim, Germany) and processed using Adobe Photoshop 6.0. We quantified the percentages by counting CD40, HLA-DR and MR positive cells in ten randomly taken pictures at magnification 20X of the active lesions.

### Statistical analysis

The data were analyzed using a one-way ANOVA with Bonferroni correction in Graphpad prism version 4.03 for Windows (Graphpad software, San Diego, California, USA). A *P* value ≤0.05 was considered significant.

## Results

### Expression of M1 and M2 markers on *in vitro* generated macrophages

**Figure 2:**
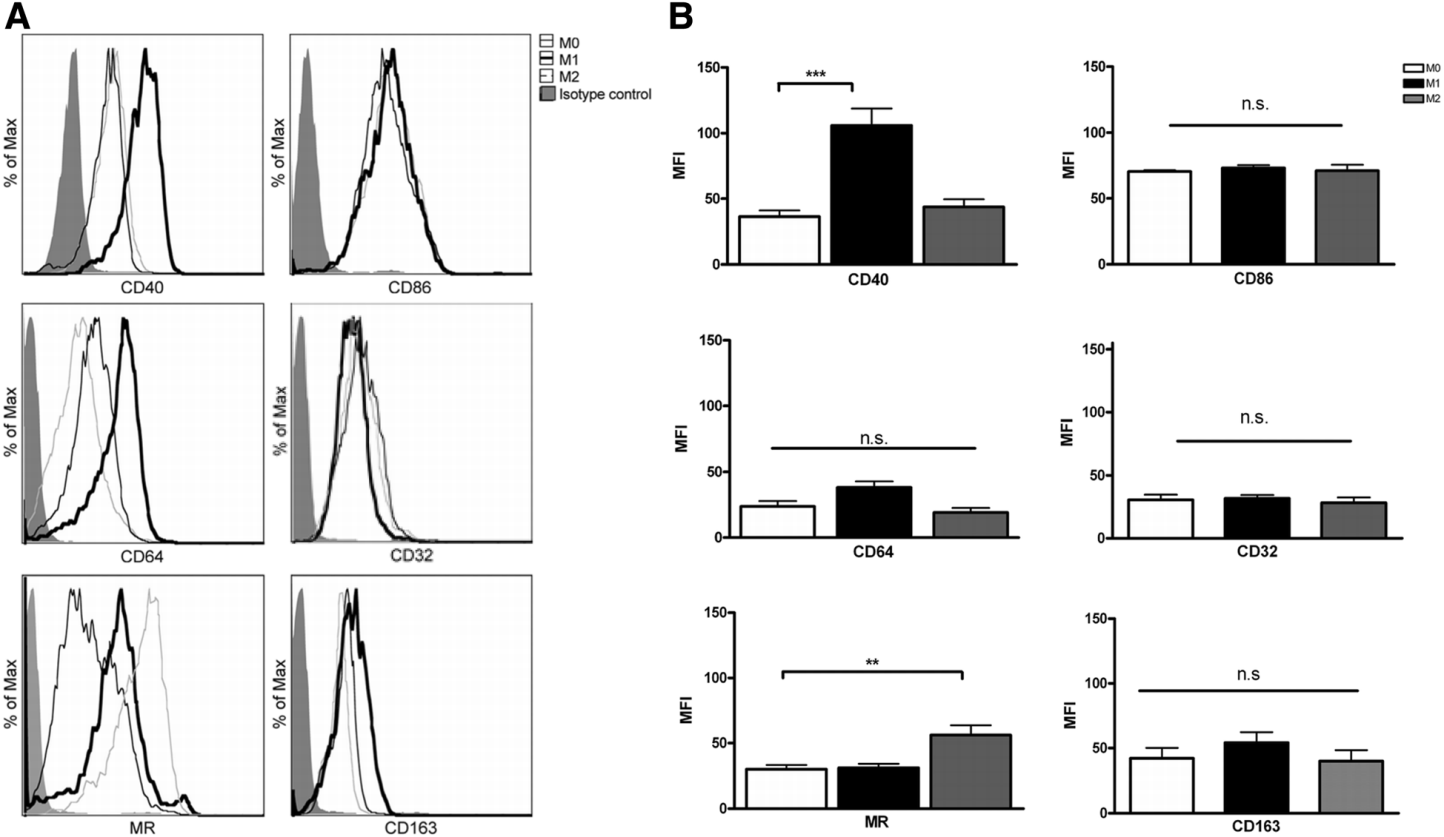
**Expression of markers on M0, M1 and M2 macrophages stimulated *in vitro.*** Macrophages were polarized to M1 and M2 macrophages (see materials and methods for details) and the expression of markers was analyzed by flow cytometry. In all figures the black bold line represents M1 macrophages (**A**). CD40 and CD64 are both shifted in mean fluorescent intensity; however, only CD40 is significantly upregulated on M1 macrophages compared to M0 macrophages. The M2 macrophage population is depicted by a grey line. Mannose receptor (MR) expression is upregulated on the M2 macrophages, whereas no differences in CD163 expression were observed comparing the different subsets. The means ± SEM were calculated from three independent experiments performed in duplicate and *P <0.05 calculated for mean fluorescent intensity (MFI) was considered significant (**B**).

PBMCs were obtained from three different donors and cultured for 7 days to mature into macrophages, then stimulated with either IL-4 (M2) or IFN-γ and LPS (M1) or left unstimulated (M0). Macrophages were harvested after 2 days and stained with 7-aminoactinomycin (7-AAD) (Invitrogen) to determine cell viability (approximately 80%). FACS analysis showed that 97% of the cells of each subpopulation were CD68 positive (Figure 1). We next assessed the phenotype of M1 and M2 human macrophages generated *in vitro* using a selection of various well-defined macrophage markers, including CD40, CD86, CD64, CD32, MR and CD163. Treatment with IFNγ and LPS resulted in a significant upregulation of CD40 compared to M0 macrophages (Figure 2A), in line with previous observations [32]. Treatment with IL-4 resulted in a significant increase of MR expression compared to M0 macrophages (Figure 2A). Remarkably, cell surface expression of the M1 markers CD86 and CD32 and the M2 marker CD163 did not differ significantly compared to M0 macrophages. CD64 expression showed a tendency towards upregulation on M1 macrophages; however, this did not reach significance. Results are represented in a graph depicting the MFI in Figure 2B. Statistically significant differences in MFI compared to M0 macrophages were observed for CD40 on M1 macrophages and MR on M2 macrophages (*P* <0.05).

### Expression of M1 and M2 macrophage markers in control brain and normal appearing white matter

**Figure 3:**
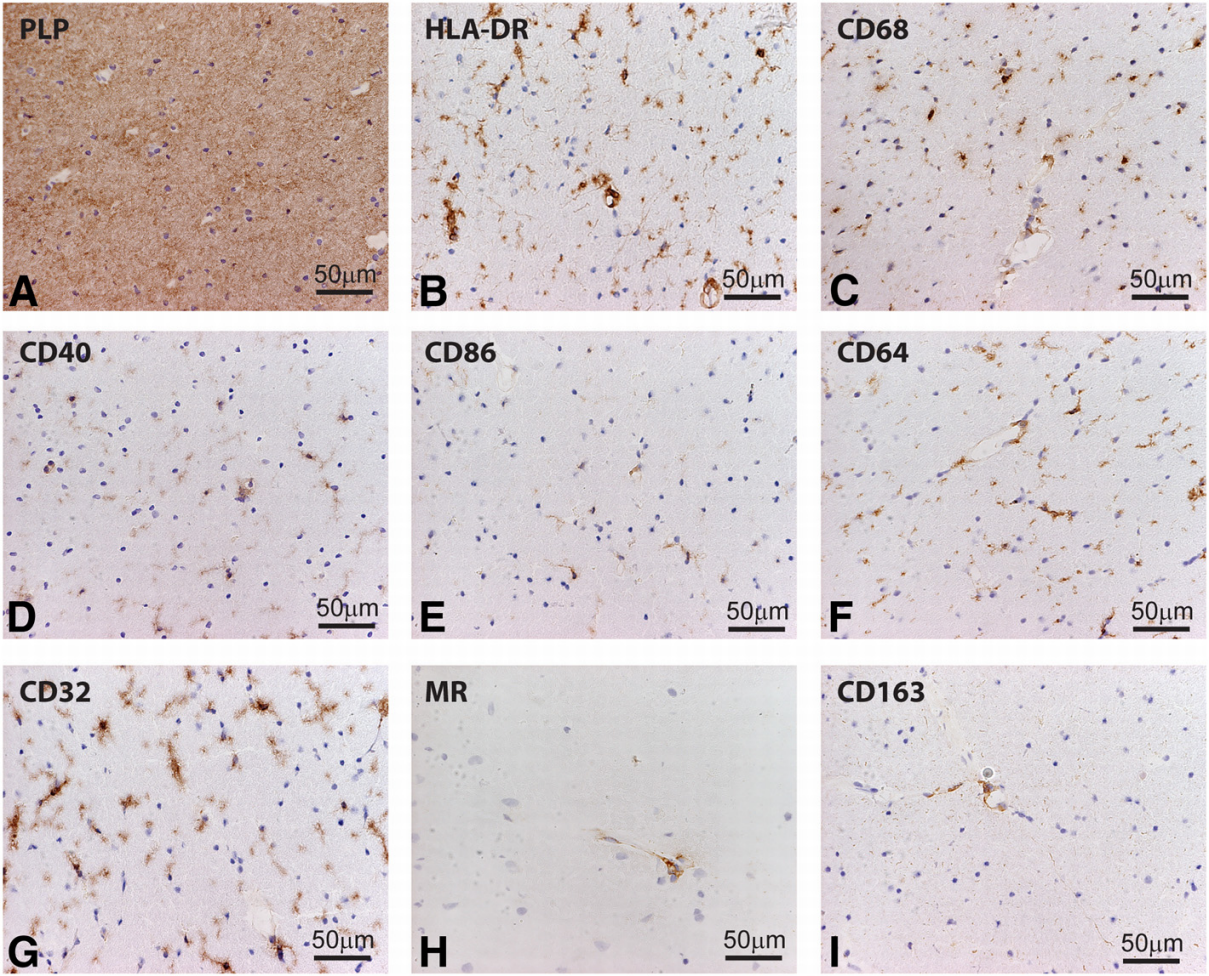
**Expression of markers for M1 and M2 phenotype in white matter of control brain.** Sections of white matter of control brain were stained using immunohistochemistry. Proteolipid protein (PLP) staining shows normal abundant myelin (**A**) Human leukocyte antigen-DR (HLA-DR) and CD68 staining reveals positive microglia (**B,C**). M1 markers, including CD40, and CD86 were expressed on microglia (**D,E**). Antibodies directed against CD64 and CD32 clearly decorated microglia (**F,G**), whereas MR and CD163 were expressed by perivascular macrophages (**H-I**).

While many studies have reported expression of typical M1 and M2 markers by macrophages this has not been systematically studied in detail in MS brain samples. To determine a baseline we first examined the expression of typical M1 and M2 markers in the brain of control subjects. CD68 and HLA-DR expression, well-known markers for macrophages and microglia, was expressed by microglia and perivascular macrophages (PVM) [42]. Likewise, antibodies directed against the M1 markers, CD40, CD64 and CD32 decorated virtually all microglia and PVM, whereas CD86 was expressed by only a minor subset of microglia. CD40 was also expressed by brain endothelial cells. As described previously, the expression of typical M2 markers MR and CD163 were restricted to PVM [47] (Figure 3). In general, M1 and M2 markers revealed a similar cellular distribution and expression pattern in NAWM compared to control white matter.

### Expression of M1 and M2 macrophage markers in MS lesions

**Figure 4:**
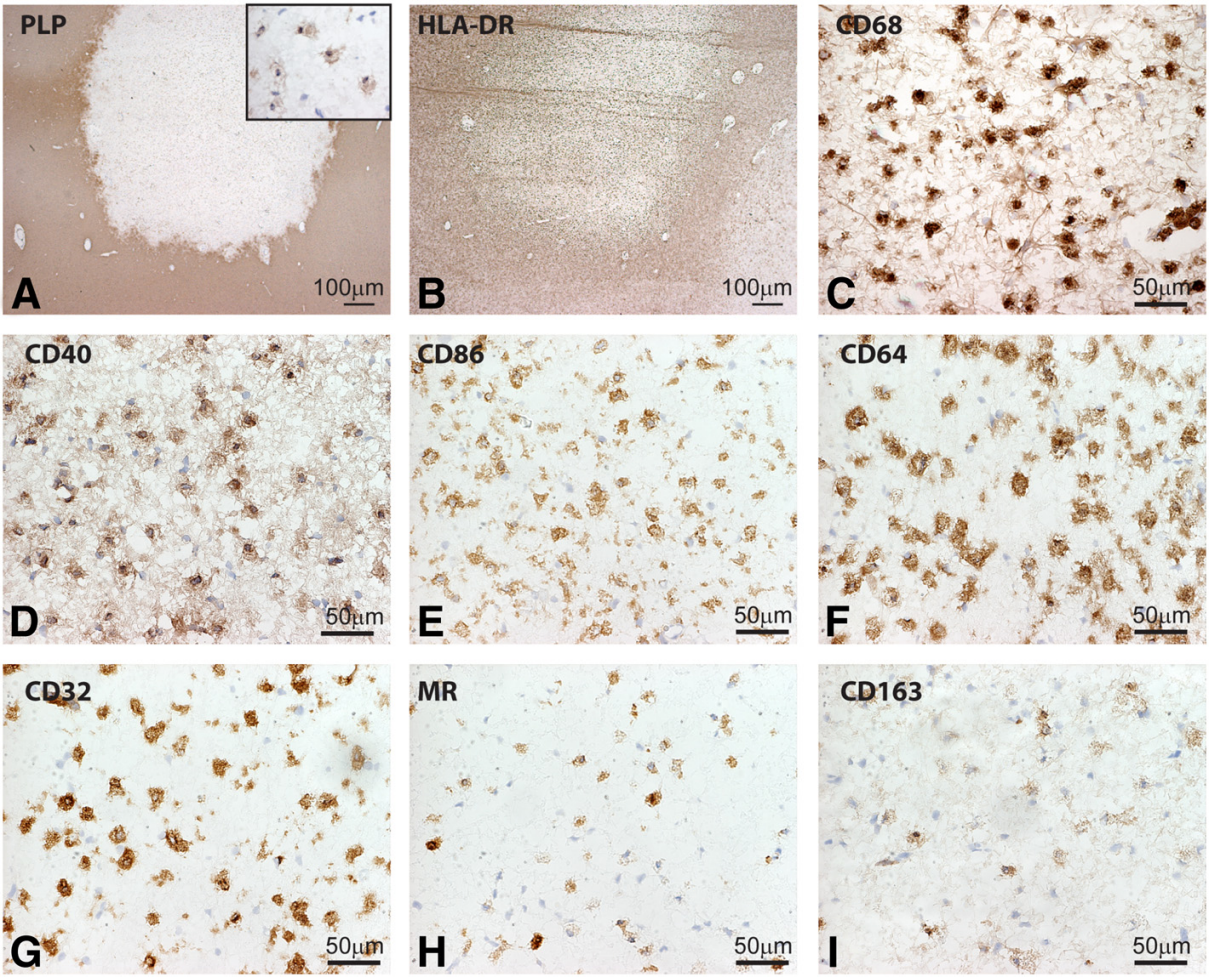
**Expression of M1 and M2 markers in active multiple sclerosis (MS) lesions.** Images were taken from the center of active demyelinating lesion. Proteolipid protein (PLP) staining shows widespread demyelination and PLP-laden macrophages (insert) (**A**). Intense labeling of human leukocyte antigen-DR (HLA-DR) and CD68 positive cells was observed in the center and rim of the lesion. CD68, CD40, CD86, CD64 and CD32 were markedly expressed by macrophages throughout the lesion area (**D-G**). Mannose receptor (MR) and CD163 were highly expressed by foamy macrophages (**H,I**).

Expression of M1 and M2 macrophages/microglia markers was evaluated in seven active and five chronic active MS lesions from eight different donors. All M1 markers studied, including CD40, CD86, CD64 and CD32, were consistently and highly expressed by activated microglia and myelin-laden macrophages throughout the demyelinated lesion area. Anti-CD40 showed both a cytoplasmic and membrane staining pattern while the Fc-γ receptors CD64 and CD32 were present on membranes of all macrophages and microglia. In active MS lesions MR and CD163, two well-defined M2 markers, were strongly expressed by foamy macrophages and by a majority of the PVM (Figure 4).

**Figure 5:**
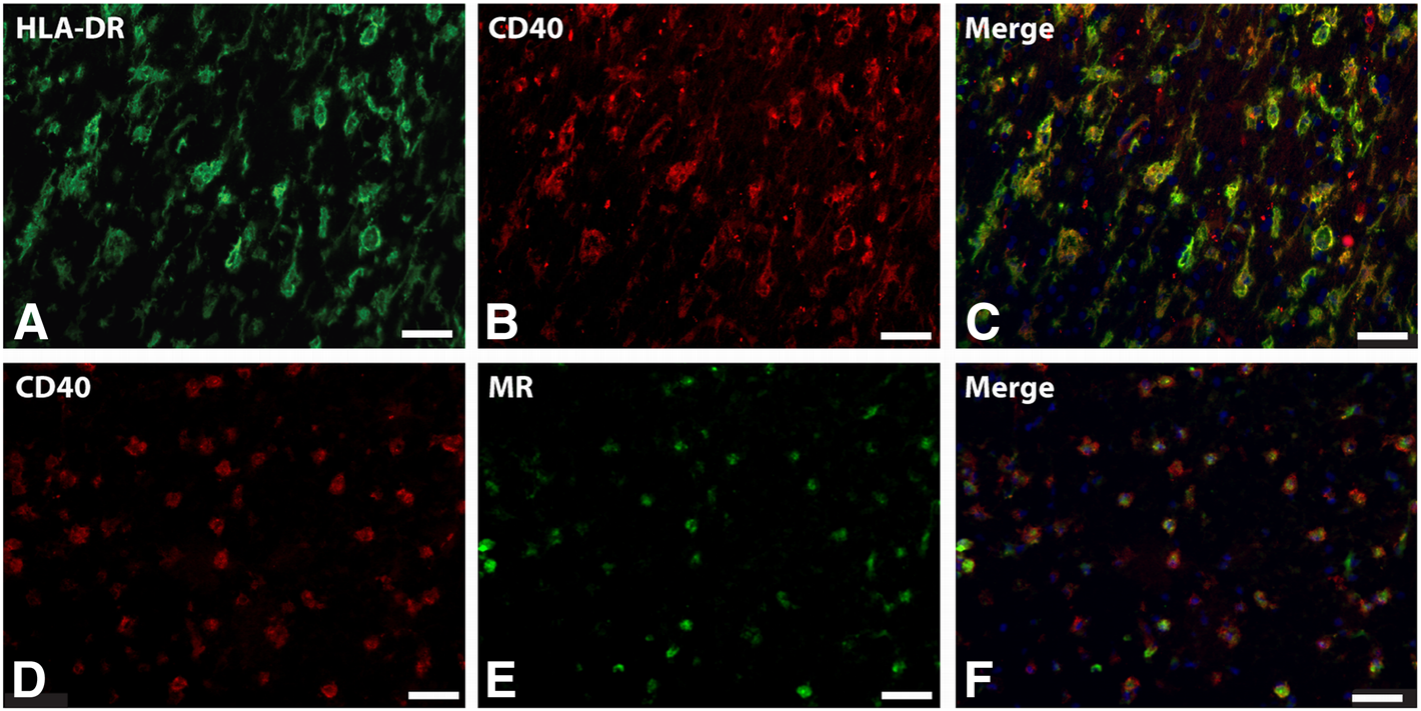
**CD40 and mannose receptor (MR) expression on foamy macrophages in an active multiple sclerosis (MS) lesion.** Images are taken at the center of an active MS lesion stained for human leukocyte antigen-DR (HLA-DR) **(A)** and CD40 **(B)**. Colocalization studies showed a clear overlap of HLA-DR and CD40 **(C)**. Double staining with anti-CD40 **(D)** and -MR **(E)** shows that 70% of the CD40 positive cells were also MR positive, all MR positive cells are CD40 positive **(F)**.

Immunofluorescent double staining on five active lesions and chronic active lesions of CD40 and HLA-DR revealed a complete overlap on macrophages/microglia, indicating that virtually all macrophages/microglia express CD40. To study the co-expression of M1 and M2 markers, double staining on both active and chronic active lesions of CD40 with MR was performed. All macrophages/microglia expressed CD40 and 70% (range 51 to 80%) of foamy macrophages expressed both MR as well as CD40 in active MS lesions (Figure 5). The overlap of M1 and M2 markers was consistently observed in lesion samples of the different patients.

**Figure 6:**
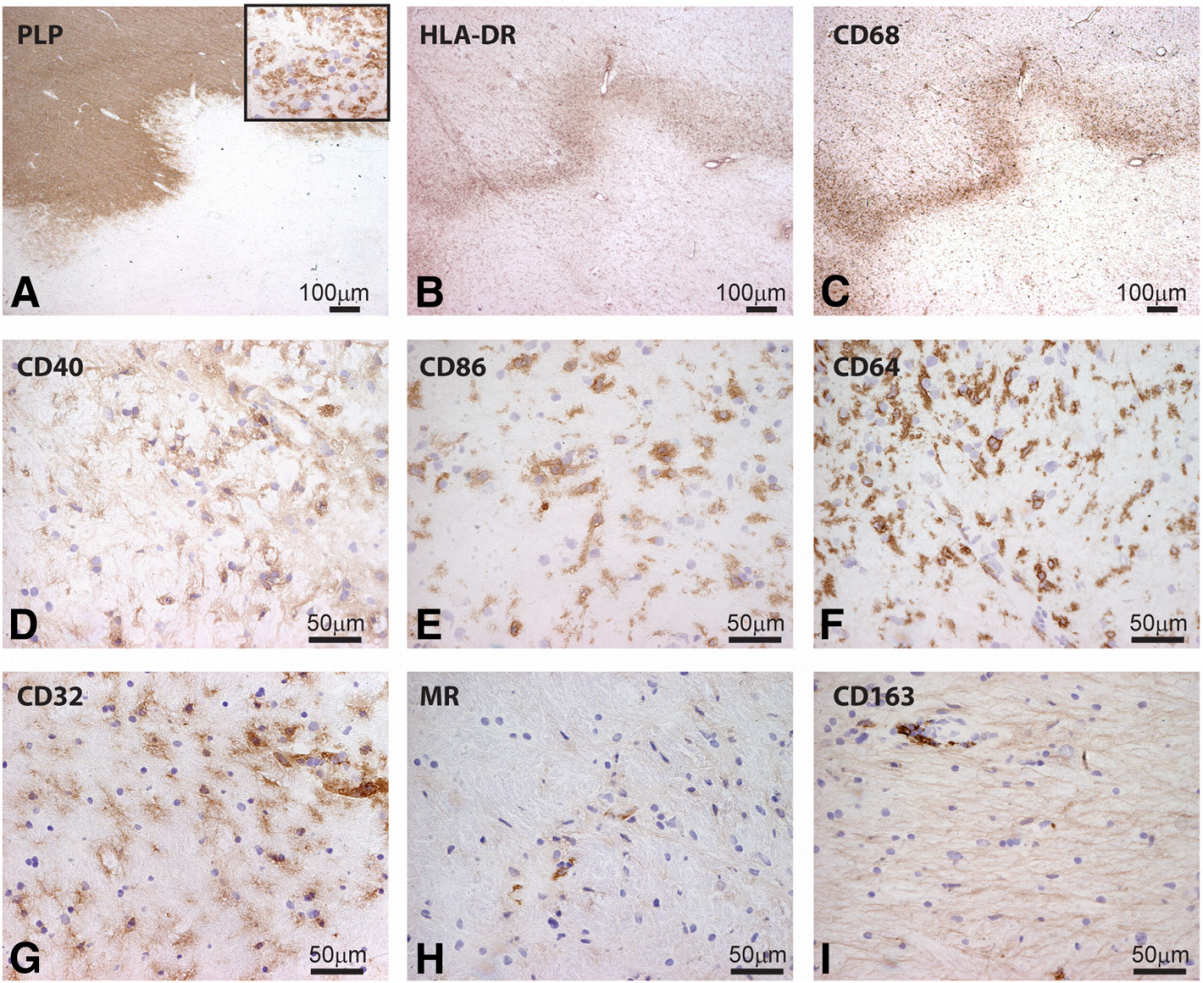
**Expression of markers for M1 and M2 phenotype in a chronic active lesion. (A)** Proteolipid protein (PLP) staining of a chronic active lesion shows massive demyelination and PLP positive macrophages in the insert. Human leukocyte antigen-DR (HLA-DR) expression was profound at the rim of chronic active lesions (**B**). CD68, CD40, CD86, CD64 and CD32 are all clearly expressed by microglia at the rim of the lesion. Images of macrophage markers were taken at the rim of the lesion (**C-G**). Mannose receptor (MR)-positive and CD163-positive macrophages were predominantly observed in the perivascular space **(H-I)**.

Chronic active lesions are characterized by a demyelinated gliotic center and hypercellular rim containing HLA-DR and CD68-immunopositive activated microglia and macrophages. In our study most chronic active lesions had only activated microglia in the rim and no foamy macrophages. Only one chronic active lesion contained foamy macrophages. All M1 markers were abundantly expressed by activated microglia at the rim of chronic active lesions (Figure 6). Activated microglia in the rim of chronic active lesions lacked MR and CD163 expression, whereas PVM prominently expressed MR and CD163 (Figure 6H,I). The results are summarized in a semi-quantification of the expression of the markers in control brain, NAWM, active MS lesions and chronic active MS lesions (Table 3).

**Table 3:** Marker expression in multiple sclerosis (MS) lesions

**Macrophage**	**Antigen**	**Control**	**NAWM**	**Active lesion**	**Chronic active lesion**
Pan marker	CD68	+	+	+++	+++
M1	CD40	+	+	+++	+++
	CD86	+	+	++	+++
	CD64	++	++	+++	+++
	CD32	++	++	++	++
M2	Mannose receptor	-	-	+++	-
	CD163	-	-	+++	-

NAWM, normal appearing white matter; ‘-‘ indicates no positive cells; ‘+’ indicates few macrophages stain positive or the expression level is low; ‘++’ indicates that the majority (>50%) of the macrophages stain positive; ‘+++’ indicates that all macrophages stain positive.

In summary, we here show for the first time that the majority of foamy macrophages in active MS lesions consistently express both M1 and M2 markers, indicating an intermediate activation status. This was a consistent finding in all lesions studied.

## Discussion

Macrophages are the most predominant immune cell type in inflammatory demyelinating MS lesions. The activation status of macrophages in MS lesions has not been studied in detail yet. Therefore, the aim of this study was to systematically analyze the expression of markers for M1 (classically activated, pro-inflammatory) and M2 (alternatively activated, growth promoting) macrophages/microglia in the different lesion types. Our findings indicate that CD40 and MR are the most distinctive markers for M1 and M2 macrophages*. In vivo* examination of the active and chronic active MS lesions revealed that the expression of typical M1 markers is more abundant than that of M2 markers and approximately 70% (51 to 80%) of foamy macrophages present in active demyelinating lesions express both M1 and M2 markers. In chronic active lesions the M2 markers are lacking, indicating that the M2 markers extinguish slowly when an active lesion is developing towards a chronic active lesion.

Many factors are proposed to push polarization besides IFNγ/LPS and IL-4, such as M-CSF and GM-CSF, which induce other phenotypic marker expression. Factors involved in cell culture, including growth factors, media, supplements and stimulation methods will influence marker expression by the subsets [32,33,48-50]. Our *in vitro* data show that CD40 is the most distinctive marker for the M1 phenotype, which is in line with other studies [34,51]. Both LPS and IFNγ (stimuli for classical activation), induce CD40 expression by macrophages and microglia via activation of NFκB [34,51]. In contrast to previous reports [28,29] we found no significant differences in the expression of CD86, CD64 and CD32 between M1 and unstimulated macrophages. In previous studies CD86 is significantly upregulated after IFNy or IL-4 stimulation compared to M0; however, it upregulated to the same extent between M1 and M2 which is in line with our data [35]. Zeyda *et al*. showed that CD86 was significantly upregulated between the subsets after stimulation with IL-4 and to a greater extent with IFNγ compared to adipose tissue macrophages [34]; however, their culture method deviates from our protocol; DNA-se was added and macrophages were cultured in RPMI/FCS10%, which may influence the marker expression. CD64 and CD32 were upregulated in a study by Becker after stimulation with IFNγ only [49]; however, another study showed no upregulation of CD32 upon IFNγ stimulation [35]. The difference in CD32 expression after IFNγ polarization can be explained by different media, different amounts of IFNγ and a different activation time scheme, RPMI with 5% NHS and IMDM with 10% FCS, 0.001-1 ng/ml IFNγ and 50 ng/ml, 24 to 64 h and 96 h, respectively. CD32 showed no difference *in vitro* using our stimulation method of combining IFNγ and LPS to skew macrophages towards an M1 phenotype. CD64 is considered a marker for M1 macrophages [35,49]; however, in a contradicting study, CD64 was not upregulated on M1 macrophages [32]. We observed that CD64 was higher expressed on M1 macrophages compared to M0 macrophages; however this higher expression did not reach significance. Our findings illustrate that the expression of MR is significantly higher on M2 macrophages after IL-4 stimulation, which is in line with previous findings [35,50,52]. In our experiments CD163, a marker that is expressed on M2 macrophages [32,35,36], was not upregulated after IL-4 stimulation as has been demonstrated before [34,50]. Altogether, our data show that IFNγ/LPS skews macrophages to CD40-immunopositive M1 macrophages, whereas IL-4 exposure promotes the induction of MR-positive M2 macrophages. Taken together these studies indicate that there is only a partial phenotypic overlap between GM-CSF and M-CSF polarized macrophages and IFNγ/LPS and IL-4 skewed macrophages.

*In vivo*, the activation status of macrophages is likely induced by a complex set of factors, which includes, but is not limited to, the commonly used *in vitro* activators. In normal appearing white matter an upregulation of possible microglia activators like IL-4, IL-1β are present, compared to control brain [53]. Because a plethora of activators is present *in vivo*, we decided to continue with a panel of relevant markers to study the presence of M1 and M2 phenotype. Our results show that microglia in NAWM and control brain express M1 markers, FC-gamma receptors, CD64 and CD32, which is line with previous findings [54]. A recently published study stressed the differences between NAWM and control brain by showing the activation status of microglia, based on marker expression of microglia on RNA level [55]. We could not confirm these findings by the expression of M1 and M2 markers at protein level. It could well be that detection at mRNA level is more sensitive than immunohistochemical detection of protein expression.

In control brain the expression of CD86 is lacking and CD40 is present on PVM, microglia and endothelium. The expression of these co-stimulatory molecules in NAWM have not been reported before in detail [47,56,57]. Here we show that CD40 is expressed by most microglial cells and endothelium, whereas CD86 is weakly expressed on the branches of a subset of microglia. No major differences in expression of CD40 and CD86 were observed between control brain and NAWM. These findings in human brain are in contrast with findings in marmoset monkeys where CD40 and CD86 were completely absent in control brain from marmoset monkeys [58]. Regarding the expression of M2 markers present in the control brain, it was shown previously that the expression of M2 markers is limited to the PVM in NAWM and control brain as confirmed in our study [38,47]. It is postulated that microglia in control brain are in a resting state [59]. However our data indicate a more ‘vigilant’ state since in NAWM and control brain the Fc-gamma receptors, CD64 and CD32 are expressed by microglia, as well as the co-stimulatory molecules CD40 and CD86.

In active MS lesions all foamy macrophages express CD40, CD86 CD64 and CD32 indicative of an M1 phenotype. Previously the expression of Fc-γ receptors, CD64 and CD32 by macrophages in active lesions and the presence of co-stimulatory molecules CD86 and CD40 is described before in the CNS on microglia [47,54,56,60]. We here show that CD86 and CD40 are not only expressed by microglia, but also by foamy macrophages, indicating their activated state. CD40 is of special interest, since this receptor is known to play a crucial role in experimental autoimmune encephalomyelitis (EAE) [58]. Interaction between CD40 on macrophages/microglia and its ligand leads to secretion of cytokines and neurotoxins and CD40-deficient mice and CD40-ligand knockout mice fail to develop EAE [61,62]. Animals with EAE treated with antibodies against CD40 showed reduced clinical signs indicating that CD40 is crucial for disease induction and neuroinflammation [41,63-65]. Presence of CD40 was described on foamy macrophages in active MS lesions and on endothelium [41,47,57]. In this study we show that CD40 is abundantly expressed by virtually all microglia cells and macrophages in MS brains, indicating the vigilant state of these HLA-DR positive cells.

The expression of M2 markers on macrophages was shown before [38,47]; however, we are the first to show that MR is also expressed on a majority of foamy macrophages and absent on activated microglia. Scavenger receptor CD163 showed a similar distribution pattern as MR, with strong expression on PVM and foamy macrophages [47,66]. Upregulation of MR and CD163 on macrophages is consistently interpreted as an anti-inflammatory macrophage activation status [30,38]. However, these receptors are both pathogen recognition receptors, suggesting that macrophages are actively involved in innate immunity [10].

Our findings confirm previous data demonstrating that HLA-DR-positive microglia almost have a complete overlap with CD40 immunoreactivity [57]. Double immunofluorescence staining revealed that approximately 70% of CD40-positive (foamy) macrophages/microglia co-express MR. Only one previous study showed that a subpopulation of PVM in an active lesion expresses both M1 and M2 markers, hinting at an intermediate activation status of the macrophage [47]. Here, we provide evidence that the majority of macrophages and activated microglial cells in active demyelinating MS lesions express a combination of typical M1 and M2 markers. This phenotype *ex vivo* differs from that found *in vitro*, which might be explained by the fact that *in vitro* stimulation methods, based on addition of either IFNγ and LPS or IL-4 does not adequately represent the *in vivo* situation where infiltrating cells are exposed to an arsenal of pro- and anti-inflammatory mediators, chemokines and growth factors.

## Conclusions

In summary, we show that CD40 and MR are the most distinctive cell surface markers to identify human M1 and M2 macrophages *in vitro*. Immunohistochemical analysis revealed that virtually all activated macrophages/microglia express the typical M1 marker CD40. Interestingly, the majority (70%) of foamy macrophages in active demyelinating MS lesions co-express M1 and M2 markers. Together, our findings suggest that, although macrophages in active MS lesions predominantly display M1 characteristics, a major subset of macrophages have an intermediate activation status. Many endogenous signals can be responsible for this intermediate activation state. We are currently investigating which factor is responsible for induction of this intermediate phenotype.

## Abbreviations

7-AAD: 7-aminoactinomycin; AA/M2: Alternatively activated; BSA: Bovine serum albumin; CA/M1: Classically activated; CNS: Central nervous system; DAB: 3,3’-diaminobenzidine; EAE: Experimental autoimmune encephalomyelitis; FACS: Fluorescence-activated cell sorting; FcγR: Fc gamma receptor; HLA-DR: Human leucocyte antigen-DR; IFN-γ: Interferon-gamma; IHC: Immunohistochemistry; iNOS: Inducible nitric oxide synthase; IL-4: Interleukin-4; LPS: Lipopolysaccharide; MFI: Mean florescent intensity; MHC Class II: Major histocompatibility complex II; MR: Mannose receptor; MS: Multiple sclerosis; NAWM: Normal appearing white matter; NHS: Normal human serum; NO: Nitric oxide; PBMC: Peripheral blood mononuclear cell; PBS: Phosphate buffered saline; PLP: Proteolipid protein; PSG: Penicillin-streptomycin-glutamine; PVM: Perivascular macrophages

## Competing interests

The authors declare that there are no competing interests.

## Authors’ contributions

EV and DV were involved in acquisition of data, data analysis and writing of the manuscript. JG and PH performed both data acquisition and analysis. MM was involved in data acquisition. PvdV provided MS brain material and was involved in data analysis. CT and SA were involved in study design. CD and JvH participated equally in study design, conceptualization, data analysis and writing of the manuscript. All authors read and approved the final manuscript.
